# Diagnostic value of RDW for the prediction of mortality in adult sepsis patients: A systematic review and meta-analysis

**DOI:** 10.3389/fimmu.2022.997853

**Published:** 2022-10-17

**Authors:** Hongsheng Wu, Biling Liao, Tiansheng Cao, Tengfei Ji, Jianbin Huang, Keqiang Ma

**Affiliations:** Hepatobiliary Pancreatic Surgery Department, Affiliated Huadu Hospital, Southern Medical University, Guangzhou, China

**Keywords:** red blood cell distribution width, sepsis, diagnostic value, mortality, prognosis, meta-analysis

## Abstract

**Background:**

Red blood cell distribution width (RDW) is a common biomarker of bacterial infections, and it can be easily obtained from a routine blood test. We investigate the diagnostic value of RDW for the prediction of mortality in adult sepsis patients through a review and meta-analysis. We registered this review in PROSPERO (Registration Number: CRD42022357712), and the details of the registration are included in Appendix 1.

**Methods:**

We searched PubMed, Cochrane Library, Springer, and Embase between Jan. 1, 2000, and May 30, 2022, for primary studies about this research. We collected articles that investigated RDW for varying degrees of sepsis patients—those who suffered from sepsis, severe sepsis, or sepsis shock. Studies of healthy people and sepsis of children and neonates were excluded from our research. The definition of study characteristics and data extraction were finished by two independent researchers and discrepancies resolved by consensus. The combined sensitivities and specificities were calculated by meta-analysis using STATA14.0. The sensitivity of the included studies was analyzed by excluding studies that had potential heterogeneity. A summary operating characteristic curve was made to evaluate the diagnostic value for the prediction of mortality in adult sepsis patients. The Fagan test was used to explore likelihood ratios and posttest probabilities. Finally, we investigated the source of heterogeneity using meta-regression.

**Results:**

Twenty-four studies, including 40,763 cases altogether, were included in this analysis. Bivariate analysis indicated a combined sensitivity of 0.81 (95% CI 0.73–0.86) and specificity of 0.65 (95% CI 0.54–0.75). The area under the summary receiver operating characteristic curve was 0.81 (95% CI 0.77–0.84). Substantial heterogeneity resided in the studies (*I^2 =^* 96.68, 95% CI 95.95–97.4). Meta-regression showed that the reference description, prospective design, and blinded interpretation of the included studies could be responsible for the heterogeneity.

**Conclusions:**

RWD is an available and valuable biomarker for prediction of mortality in adult sepsis patients.

**Systematic review registration:**

https://www.crd.york.ac.uk/PROSPERO/, identifier CRD42022357712.

## Introduction

Sepsis is one of the most serious diseases, and it threatens human health. It represents an abnormal immune reaction to infection, which leads to organ dysfunction (1–3). Up to now, the pathogenesis of sepsis has not been understood sufficiently. The currently accepted theory of its pathogenesis is that once endo- or exotoxins are released by the pathogen, immune cell receptors may activate a series of signaling pathways, which release pro- and anti-inflammatory mediators, such as acute-phase proteins, cytokines, chemokines. Under normal physiological conditions, exotoxins plays an important role in the body’s defense against pathogenic microorganisms; however, the excessive release of cytokines and the consequent chain reaction could cause endothelial damage and intestinal permeability. If prompt and effective treatment is not carried out, it may lead to systemic inflammatory response syndrome, organ dysfunction, or even sequential multiple organ failure, which increase patient mortality during hospitalization significantly (4, 5). Early identification and timely therapy are critical for the treatment of sepsis. Therefore, approaches to identifying sepsis and estimating its prognosis are developing (4, 6, 7). Red blood cell distribution width (RDW) is a common erythrocyte index, which we can easily obtain from a routine blood test. Its inexpensive characteristic and low technical requirements mean that it can be easily accessible in most medical institutions. Research indicates that RDW is an powerful biomarker for predicting some clinical illnesses, such as cardiovascular diseases (8), respiratory disease (9), hepatitis B (10), or acute appendicitis (11). For the study of the relationship between RDW and sepsis morbidity and mortality, several studies show that RDW not only has a diagnostic value, but also has noteworthy prediction of mortality for sepsis (12–14).

Recently, a meta-analysis about the prognostic role of RDW in sepsis indicated that patients with increased RDW are more likely to have higher mortality (7). However, the limitations of this research were territorial and potential publication bias. Therefore, more scientific, rigorous, and multicenter studies about the prognostic role of RDW in sepsis need to performed, and our understanding of RDW needs to be developing continually.

On the base of previous studies, we did a meta-analysis about the diagnostic value of RDW for the prediction of mortality in adult sepsis patients and did further investigation about the clinical diagnostic value of RDW.

## Methods

### Search strategy and selection criteria

We searched PubMed, Cochrane Library, Springer, and Embase for studies that involved RDW for the prediction of mortality in adult sepsis patients. The following MeSH terms and their combinations were searched: “(red blood cell distribution width OR RDW) and (sepsis OR “severe sepsis” OR “sepsis shock”) and (prognosis OR mortality).” The time period of our search was from Jan. 1, 2000, to May 30, 2022.

To ensure the rigorousness of the literature search, the reference standard for sepsis was defined based on the Third International Consensus Definitions for Sepsis and Septic Shock (Sepsis-3) (15) or SCCM/ESICM/ACCP/ATS/SIS International Sepsis Definition Conference (Sepsis-2) (16). For comparison of the research, a positive result was defined as the sepsis patient who had an ending of death or no survival during the observation period, and the comparators for the analyzed studies were a negative result, defined as sepsis patient who had a survival ending. Furthermore, a 2×2 contingency table should be provided from the studies, and if not available for 2×2 contingency, the receiver operating characteristic (ROC) curve about sensitivity and specificity prediction of sepsis mortality, the numbers on survival and nonsurvival, must be indicated so that we can convert the data mentioned above into true positive, false positive, true negative, and false negative statistics. Only adult patients who suffered from sepsis were included in our research; healthy people and sepsis about children and neonates were excluded.

### Procedures

Two researchers (Hongsheng Wu and Biling Liao) extracted the data from the included studies independently. When a discrepancy occurred, a consensus meeting was held to resolve the problem. The evaluation scale of QUADAS was used for assessing the methodological quality of the included studies (17). The 14 quality evaluation criteria included the following: Q1. Was the spectrum of patients representative of the patients who will receive the test in practice? Q2. Were selection criteria clearly described? Q3. Is the reference standard likely to correctly classify the target condition? Q4. Is the time period between the reference standard and index test short enough to be reasonably sure that the target condition did not change between the two tests? Q5. Did the whole sample or a random selection of the sample receive verification using a reference standard of diagnosis? Q6. Did patients receive the same reference standard regardless of the index test result? Q7. Was the reference standard independent of the index test (i.e., the index test did not form part of the reference standard)? Q8. Was the execution of the index test described in sufficient detail to permit replication of the test? Q9. Was the execution of the reference standard described in sufficient detail to permit its replication? Q10. Were the index test results interpreted without knowledge of the results of the reference standard? Q11. Were the reference standard results interpreted without knowledge of the results of the index test? Q12. Were the same clinical data available when test results were interpreted as would be available when the test was used in practice? Q13. Were uninterpretable/intermediate test results reported? Q14. Were withdrawals from the study explained? We used “yes,” “no,” or “unclear” as the risk of quality assessment.

### Statistical analysis

According to the sepsis patient death or not, we calculated true positives, false positives, true negatives, and false negatives of sepsis from the included studies and figured out the sensitivity and specificity and their 95% CI.

The “MIDAS” module was used for synthesizing the data to explore the combined sensitivity and specificity and their 95% CI. The “metaninf” order was used for evaluating the sensitivity of the data synthesis. The summary ROC (SROC) was used for calculating the area under the curve (AUC) of the diagnostic value. A Fagan plot showed the relationship among prior probability, likelihood ratio, and posterior probability. Finally, a funnel plot was used for investigating the publication bias, and meta-regression was used to explore the source of heterogeneity from the included studies. All of the statistical and graphical methods mentioned above were finished by STATA version 14.0.

## Results

### Literature selection and quality assessment

**Figure 1:**
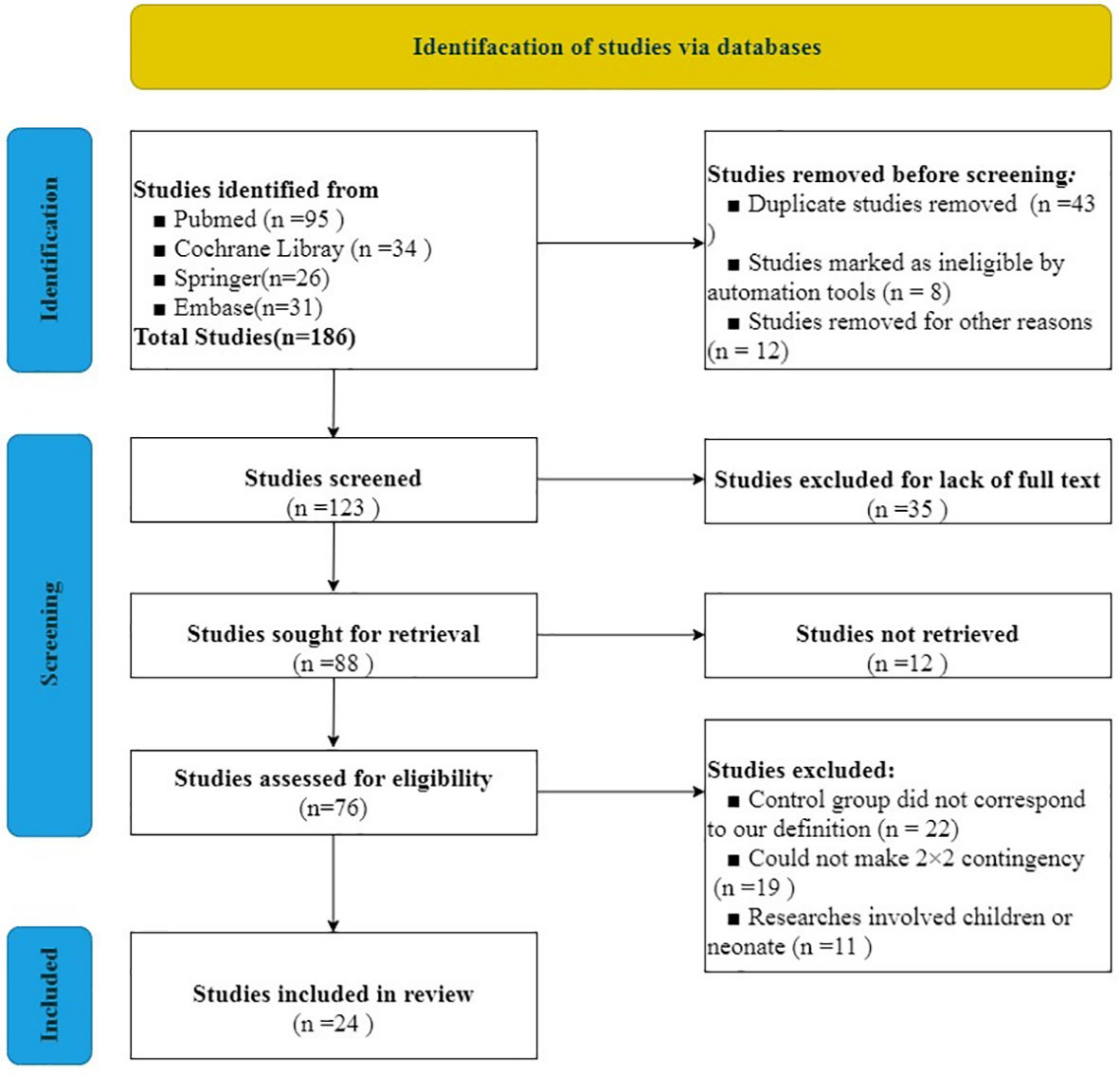
PRISMA Flowchart for selection of studies included in the systematic review.

**Figure 2:**
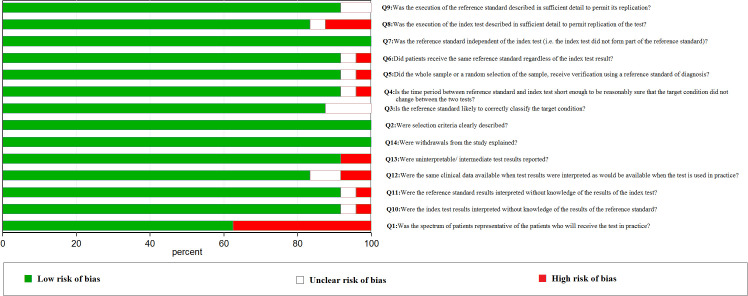
Quality assessment of the including studies base on QUADAS quality evaluation scale.

We used the PRISMA 2020 (18) statement to finish the selection of included studies. After evaluating and screening carefully from all of the studies from the databases, 24 studies including 40,763 cases were included in our analysis. The literature screening process is indicated in **Figure 1**. Literature quality assessment was estimated by QUADAS (17). We made great efforts to score each item according to this scale and how the items were assessed. The results of the included studies’ quality assessment is shown in **Figure 2**.

### Characteristics of the included studies

From all of the included studies, the severity of 15 studies (19–33) was defined as “sepsis,” eight studies (13, 34–40) were defined as “sepsis or sepsis shock,” and one study (41) was defined as “severe sepsis.” Twenty-one studies (13, 19–22, 24–36, 39–41) explicitly mentioned the cutoff value of RDW, and the other three studies (23, 37, 38) had no mention about the cutoff value of RDW. The cutoff value of RDW ranged from 13.3 to 17.0 in the included studies that mentioned it. For the mortality time, the longest observation time published was 90 days in just one study (30), yet the shortest observation time published was 1 day (35). Eight studies (19, 21, 23, 28, 31–33, 37) did not provide a definite mortality time and just defined it as “during ICU hospitalization” or “during hospitalization.” The characteristics of the included studies are shown in **Table 1**.

**Table 1:** Characteristics of the including studies.

Author	Year	Country	Severity	Definition	Cut-off value	Mortality time	TP	FP	FN	TN	Sensitivity (95%CI)	Specificity (95%CI)
Ghimire, R (34)	2020	Nepal	sepsis,sepsis shock	Sepsis-3	15.05	Day28	29	43	11	65	0.73 (0.56-0.85)	0.60 (0.50-0.69)
Chen, C.K (19)	2016	.China	sepsis	Sepsis-2	17	During hospitalization	474	656	3	5840	0.99 (0.98-1.0)	0.90 (0.89-0.91)
Dankl, D (21)	2022	Australia	sepsis	Sepsis-3	14	During hospitalization	1153	8144	382	6744	0.75 (0.73-0.77)	0.45 (0.44-0.46)
Jo, Y.H (22)	2013	Korea	sepsis	Sepsis-2	15.8	Day28	138	230	26	172	0.84 (0.78-0.89)	0.43 (0.38-0.48)
Wang, T.H (20)	2021	.China	sepsis	Sepsis-3	14.5	Day30	93	198	44	169	0.68 (0.59-0.76)	0.46 (0.41-0.51)
Krishna, V (23)	2021	USA	sepsis	Sepsis-3	Not mention	During hospitalization	12	18	5	25	0.71 (0.44-0.90)	0.58 (0.42-0.73)
Sadaka, F (35)	2013	USA	sepsis,sepsis shock	Sepsis-2	15.5	Day 1	63	6	31	179	0.67 (0.57-0.76)	0.97 (0.93-0.99)
Kim, S (36)	2015	Korea	sepsis,sepsis shock	Sepsis-2	14	Day30	63	119	44	232	0.59 (0.49-0.68)	0.66 (0.61-0.71)
Wang, H (24)	2021	China	sepsis	Sepsis-3	14.5	Day30	214	387	104	456	0.67 (0.62-0.72)	0.54 (0.51-0.57)
Özdoğan,H.K (25)	2015	Turkey	sepsis	Sepsis-2	16	Day7	49	12	3	39	0.94 (0.84-0.99)	0.76 (0.63-0.87)
Fontana, V (37)	2017	Belgium	sepsis,sepsis shock	Sepsis-3	Not mention	During hospitalization	35	39	17	31	0.67 (0.53-0.80)	0.44 (0.32-0.57)
Jiang, Y (26)	2019	China	sepsis	Sepsis-3	13.7	Day28	76	56	12	54	0.86 (0.77-0.93)	0.49 (0.39-0.59)
XueFeng Ju (38)	2017	China	sepsis,sepsis shock	Sepsis-3	Not mention	Day7	15	10	4	16	0.79 (0.54-0.94)	0.62 (0.41-0.80)
Gupta, M.K (39)	2020	India	sepsis,sepsis shock	Sepsis-3	16	Day3	41	1	3	15	0.93 (0.81-0.99)	0.94 (0.70-1.00)
Wen, K (27)	2022	China	sepsis	Sepsis-3	14.05	Day28	44	32	15	130	0.75 (0.62-0.85)	0.80 (0.73-0.86)
Li, Y (28)	2021	China	sepsis	Sepsis-2	14.5	During hospitalization	1304	5221	319	2899	0.80 (0.78-0.82)	0.36 (0.35-0.37)
Chan Ho Kim (13)	2013	Korea	sepsis,sepsis shock	Sepsis-s	14	Day28	23	77	10	219	0.70 (0.51-0.84)	0.74 (0.69-0.79)
Lorente, L (29)	2014	Spain	sepsis	Sepsis-2	15.5	Day30	55	70	49	123	0.53 (0.43-0.63)	0.64 (0.57-0.71)
Jandial, A (41)	2017	India	severe sepsis	Sepsis-3	14.5	Day31	108	72	6	14	0.95 (0.89-0.98)	0.16 (0.09-0.26)
Havens, J.M (30)	2018	USA	sepsis	Sepsis-3	13.3	Day90	36	185	3	151	0.92 (0.79-0.98)	0.45 (0.40-0.50)
Dubey, A (31)	2021	India	sepsis	Sepsis-2	15.9	During hospitalization	224	42	31	1003	0.88 (0.83-0.92)	0.96 (0.95-0.97)
Ling, J (40)	2021	China	sepsis,sepsis shock	Sepsis-3	16.05	Day28	159	105	68	139	0.70 (0.64-0.76)	0.57 (0.50-0.63)
Huang, Y (32)	2022	China	sepsis	Sepsis-3	13.5	During hospitalization	65	210	20	233	0.76 (0.66-0.85)	0.53 (0.48-0.57)
Song, K (33)	2022	China	sepsis	Sepsis-3	15.45	During hospitalization	20	52	12	115	0.63 (0.44-0.79)	0.69 (0.61-0.76)

### Results of combined sensitivity, combined specificity

**Figure 3:**
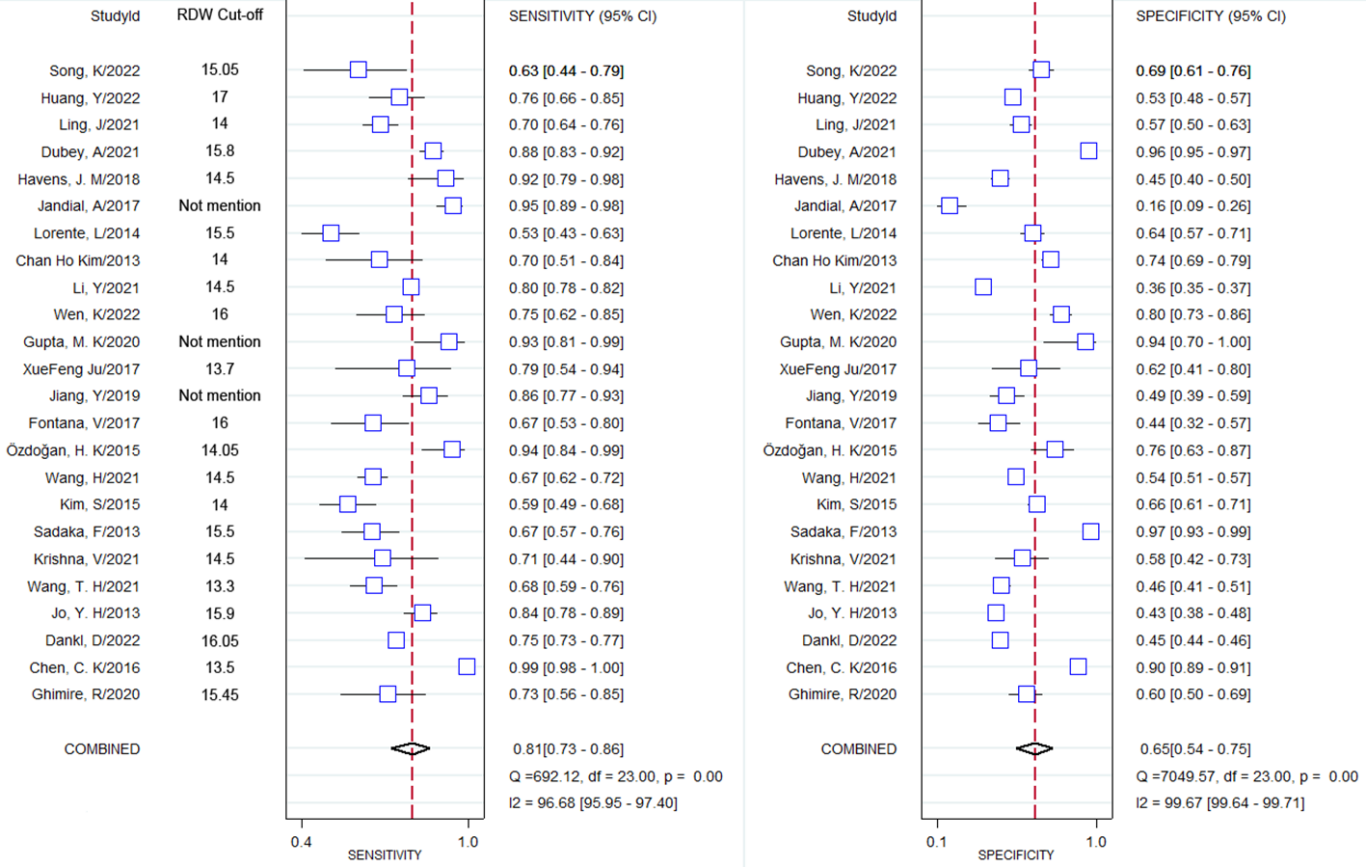
Forest plot of combined sensitivity and combined specificity and their corresponding 95% Cl.

**Figure 4:**
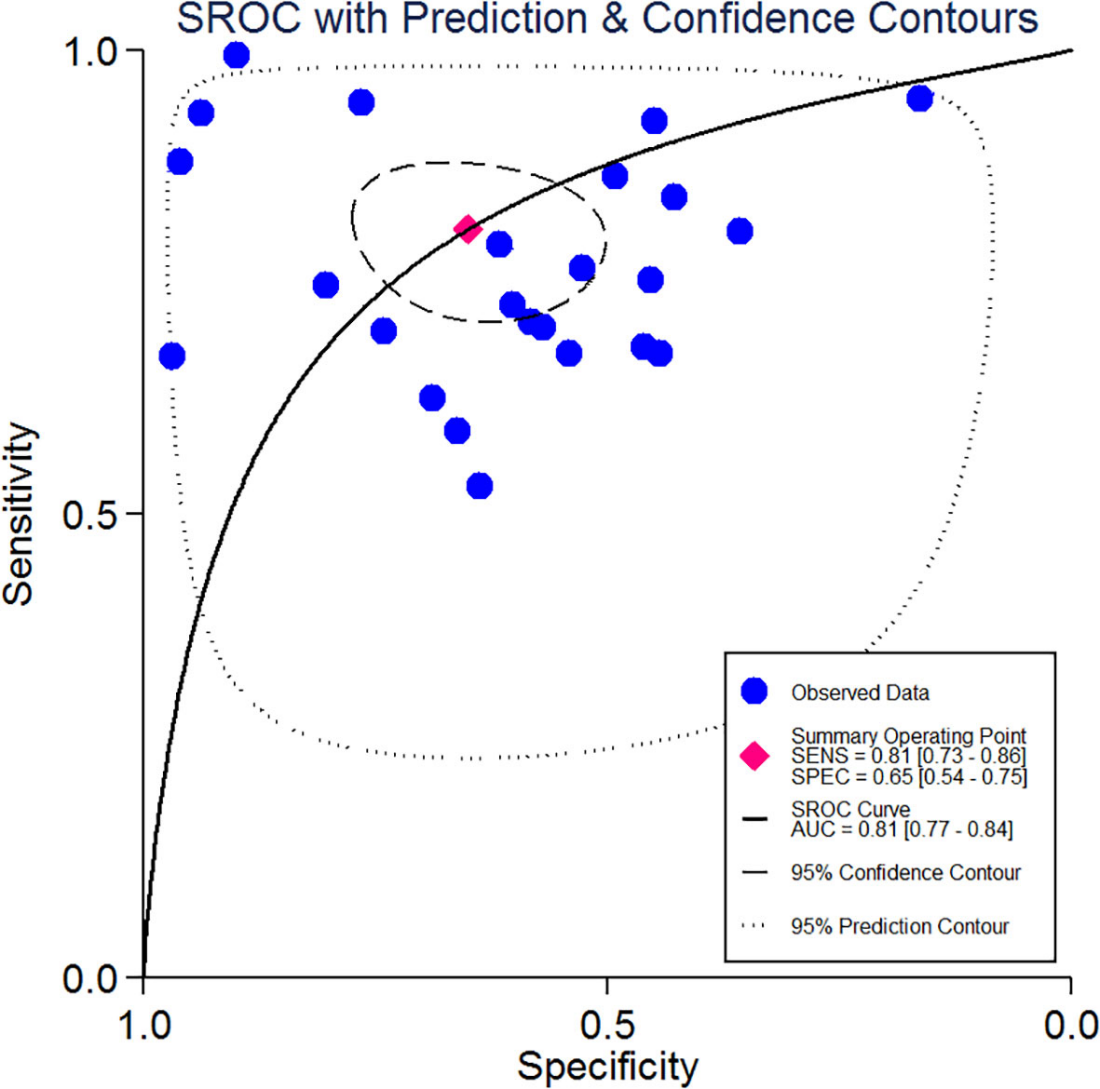
Summary receiver operating characteristics curve and the 95% confidence contour and 95% prediction contour.

Through pooling all of the included studies together through meta-analysis, the combined sensitivity was 0.81 (95% CI 0.73–0.86), and the combined specificity was 0.65 (95% CI 0.54–0.75). The result of the pooled studies is shown in **Figure 3**. To explore the diagnostic value, the SROC was made, and the results indicate that AUC and its corresponding 95% CI was 0.81 (95% CI 0.77–0.84; **Figure 4**).

### Sensitivity analysis of the pooled studies

**Figure 5:**
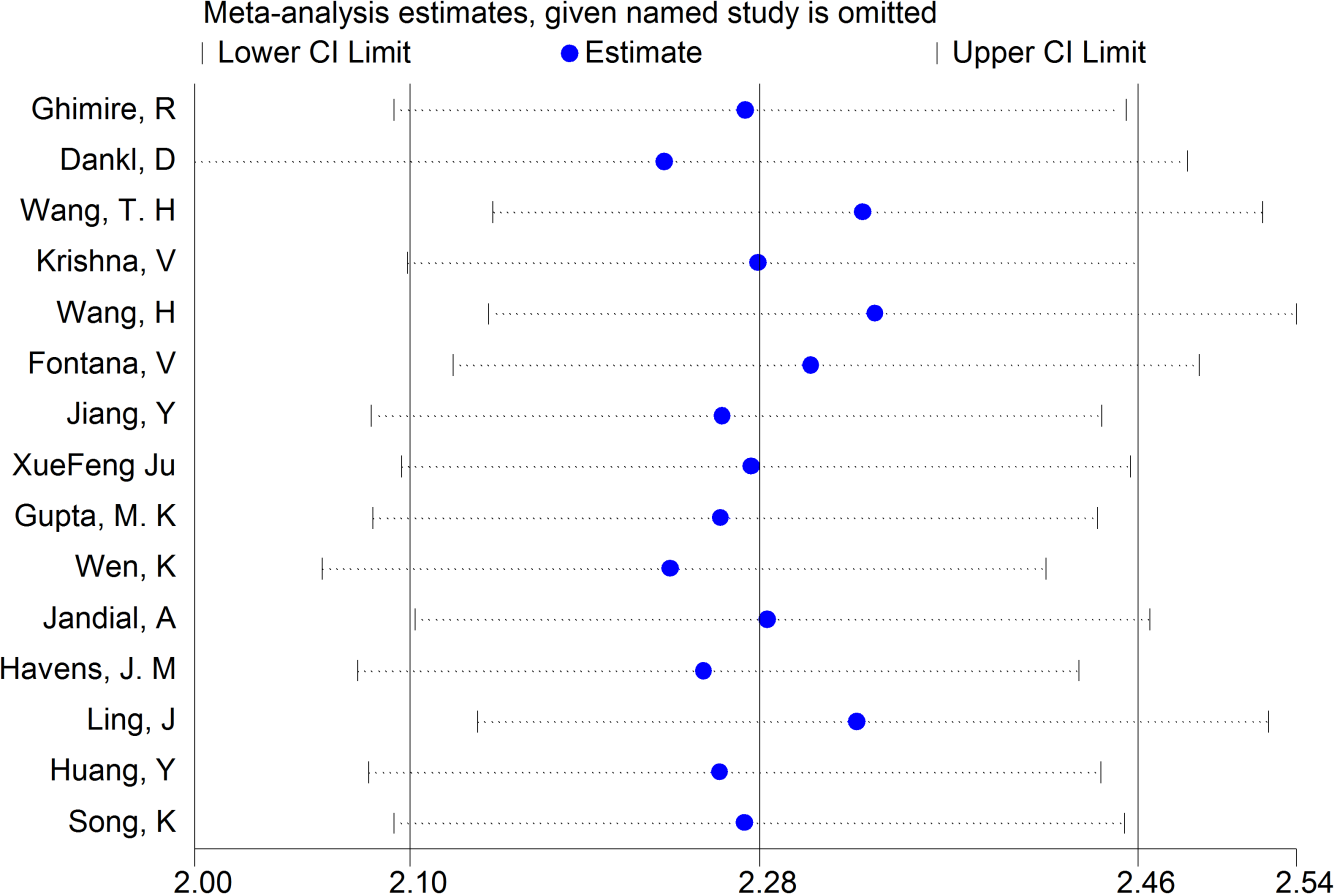
Sensitivity analysis of the result base on the new definition and clinical criteria for sepsis (Sepsis-s and Sepsis-3). Also showed the lower and upper 95% Cl.

Based on the revision of Sepsis-2 (publication in 2001), a new definition and new assessing clinical criteria for sepsis (Sepsis-3) was proposed and reached a consensus in 2016 (15, 42). According to which of the included studies are using the Sepsis-2 definition and which the Sepsis-3 definition, we also made a sensitivity analysis of this research, the aim of which was to investigate the stability of the results. We did sensitivity analysis using “METANINF.” Nine studies (13, 19, 22, 25, 28, 29, 31, 35, 36) that were defined as Sepsis-s and had potential influence on the results were excluded after removing the above four studies that had significantly impacted on sensitivity. The rest of the included studies were distributed between upper and lower 95% confidence intervals with the estimation line as the center. The sensitivity analysis is shown in **Figure 5**.

### Fagan test and Deek’s test

**Figure 6:**
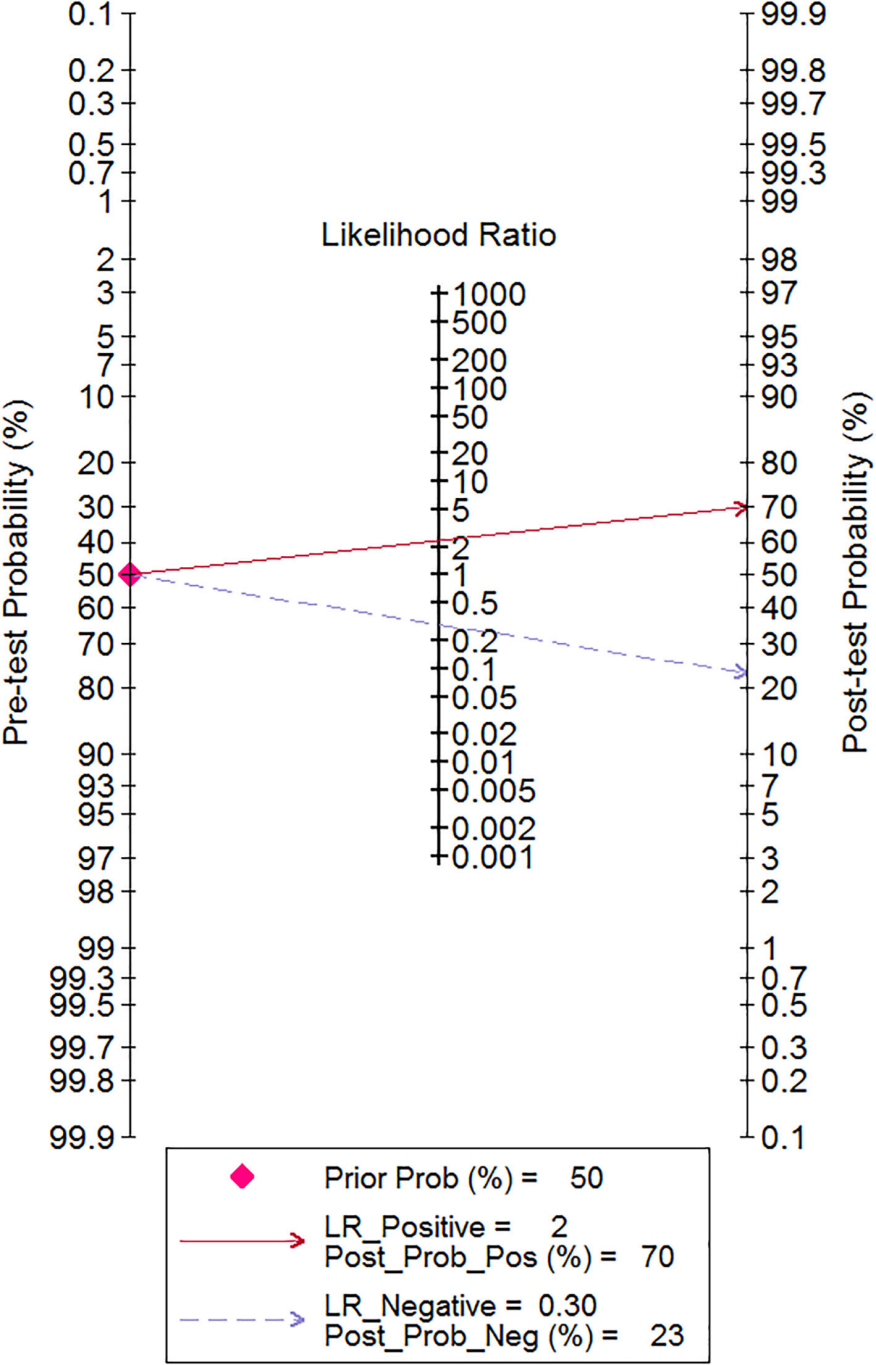
Fagan nomogram of the RDW test for diagnostic prediction of mortality in adult sepsis.

**Figure 7:**
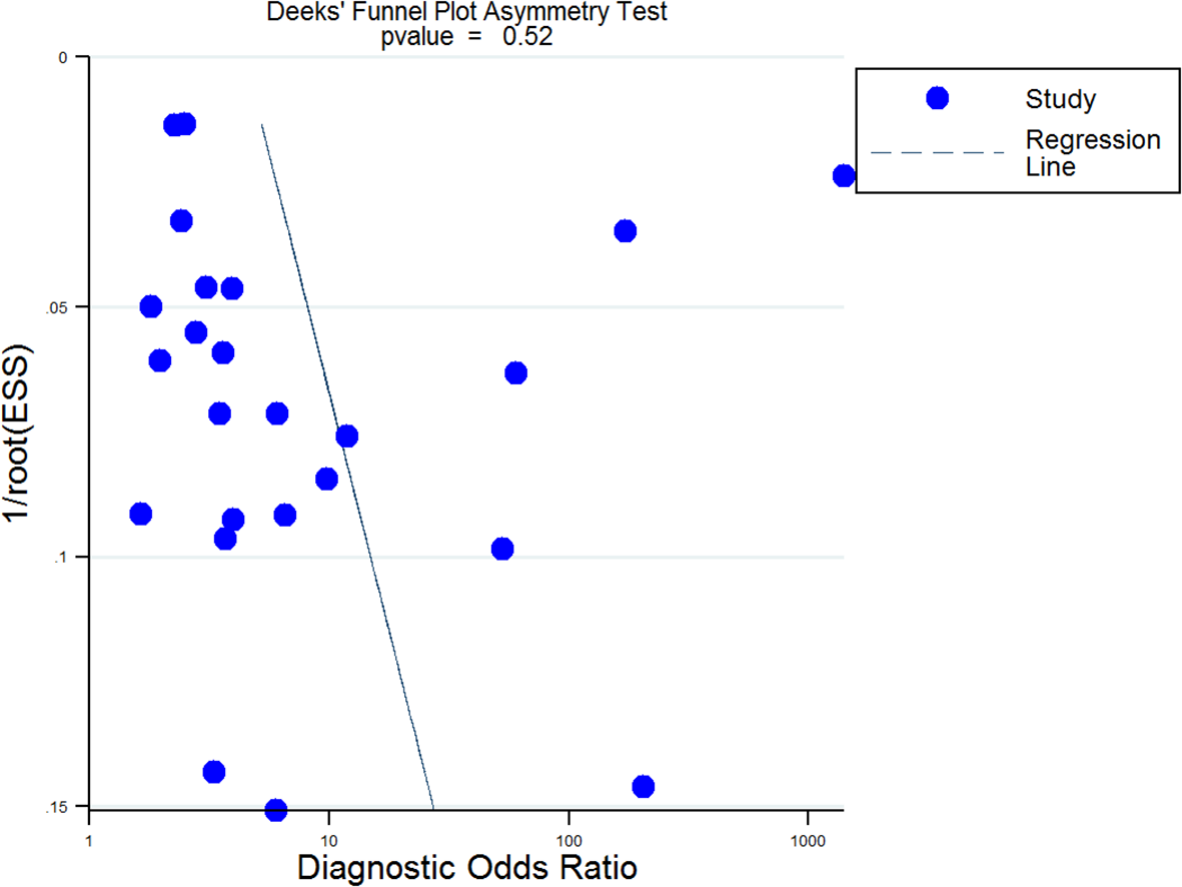
Deek’s funnel plot for publication bias analysis. A p value=0.52 suggested that the publication bias of this Meta-analysis was low. EES, Effective Sample Size.

We also used the Fagan test to evaluate likelihood ratios and posttest probabilities. The results of our research show that both likelihood ratio and posttest probability were moderate, which means that, if a sepsis patient had a high-level RDW, the prediction of mortality was 70%. Likewise, if a sepsis patient had a lower level RDW, the possibility of mortality was just 23% (**Figure 6**). For the publication bias of this meta-analysis, the funnel plot in **Figure 7** indicates that the publication bias was low (*p* value=.52).

### Heterogeneity exploration

**Figure 8:**
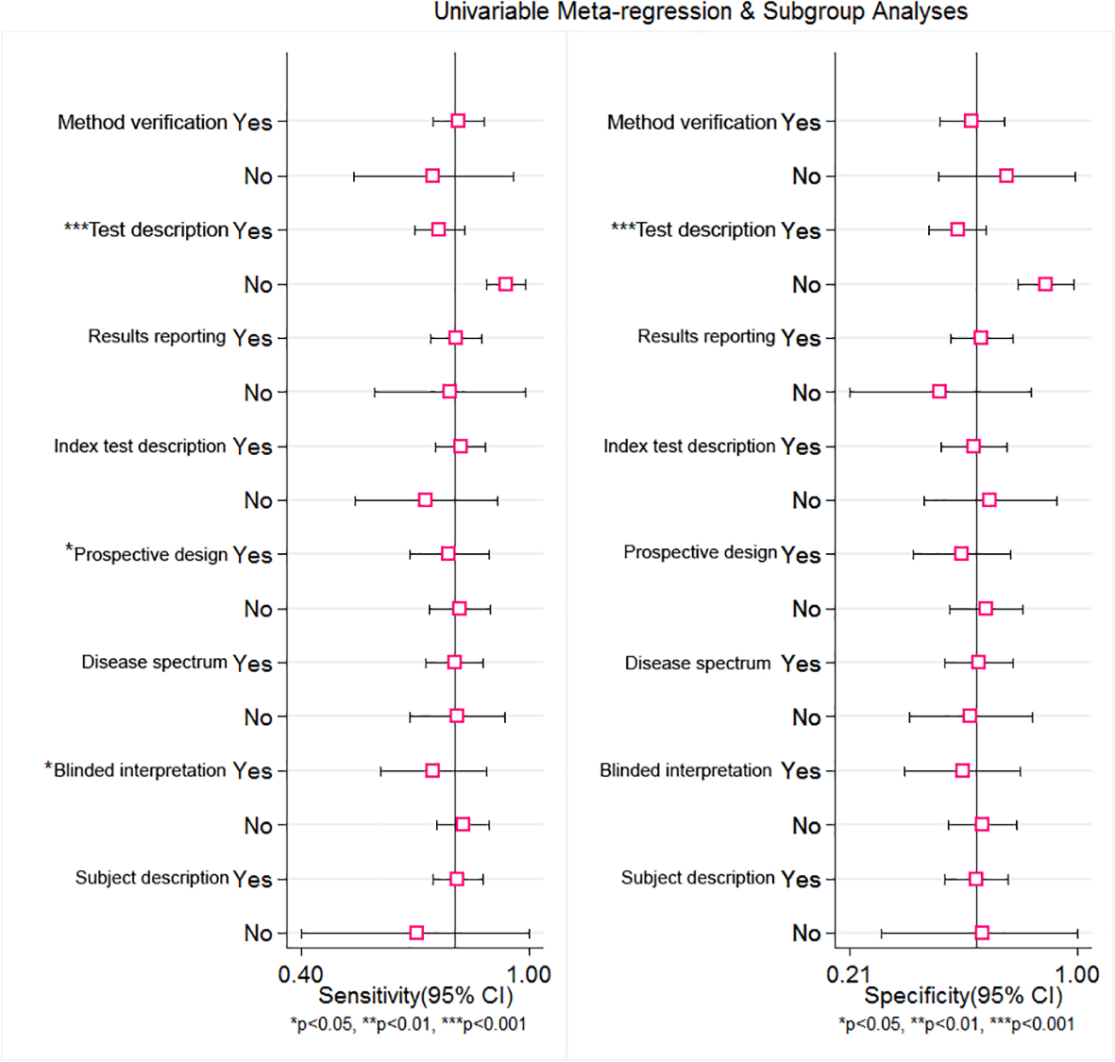
Univariable Meta-regression and subgroup analysis of RDW for predicting of mortality in adult sepsis patients. The forest plot indicated that Test description, Prospective design and Blinded may be responded for the heterogeneity.

To explore the source of heterogeneity, meta-regression was employed. From the subgroup of the sensitivity analysis, the results indicate that reference description (sensitivity: 0.76, 95% CI: 0.70–0.83, *P*=.00), prospective design (sensitivity: 0.79, 95% CI: 0.69–0.89, *P*=.03), and the blinded interpretation of the study (sensitivity: 0.75, 95% CI: 0.61–0.89, *P*=.02) could be responsible for the heterogeneity. Nevertheless, from the subgroup of the specificity analysis, only the reference description (specificity: 0.58, 95% CI: 0.48–0.68, *P*=.00) was a significant factor affecting heterogeneity (**Table 2**). The forest plot of univariable meta-regression of subgroup analysis is shown in **Figure 8**.

**Table 2:** The result of uni-variable Meta-regression of subgroup analysis.

Parameter	Category	Number of studies	Sensitivity[95%CI]	*P*_Sensitivity_	Specificity[95%CI]	*P*_Specificity_
**Method verification**	Yes	21	0.81 [0.75 - 0.88]	0.76	0.63 [0.52 - 0.75]	0.29
	No	3	0.75 [0.54 - 0.96]		0.75 [0.52 - 0.99]	
**Test description**	Yes	20	0.76 [0.70 - 0.83]	0.00	0.58 [0.48 - 0.68]	0.00
	No	4	0.94 [0.89 - 0.99]		0.89 [0.79 - 0.99]	
**Results reporting**	Yes	21	0.81 [0.74 - 0.88]	0.54	0.67 [0.56 - 0.77]	0.58
	No	3	0.79 [0.59 - 0.99]		0.52 [0.21 - 0.84]	
**Index test description**	Yes	20	0.82 [0.76 - 0.89]	0.73	0.64 [0.52 - 0.75]	0.44
	No	4	0.73 [0.54 - 0.92]		0.70 [0.46 - 0.93]	
**Prospective design**	Yes	10	0.79 [0.69 - 0.89]	0.03	0.60 [0.43 - 0.77]	0.21
	No	14	0.82 [0.74 - 0.90]		0.68 [0.56 - 0.81]	
**Disease spectrum**	Yes	18	0.80 [0.73 - 0.88]	0.10	0.66 [0.54 - 0.78]	0.74
	No	6	0.81 [0.69 - 0.94]		0.63 [0.42 - 0.84]	
**Blinded interpretation**	Yes	7	0.75 [0.61 - 0.89]	0.02	0.60 [0.40 - 0.80]	0.33
	No	17	0.83 [0.76 - 0.90]		0.67 [0.55 - 0.79]	
**Subject description**	Yes	22	0.81 [0.75 - 0.88]	0.74	0.65 [0.54 - 0.76]	0.82
	No	2	0.71 [0.40 - 1.00]		0.67 [0.32 - 1.00]	

## Discussion

Sepsis is a serious clinical syndrome caused by abnormal host immune response to infections, which may lead to organ failure or mortality (3, 4). In an estimate based on research data, the incidence of sepsis and severe sepsis were 31 and 19 million, respectively, each year and lead to about 5 million deaths annually (43). One recent review published in 2021 indicates that, from the epidemiologic analysis of the global incidence of sepsis, hospital-treated adult sepsis was 189 cases per 100,000 persons from high-income countries (44). Nevertheless, if applying the Global Burden of Diseases, Injuries, and Risk Factors Study as the reference to compare with sepsis incidence, the incidence of sepsis can be as high as 677.5 cases per 100,000 persons worldwide (45). Previous research shows that RDW is a useful prediction of mortality in adult sepsis patients (46, 47). In a meta-analysis from 2020, the result of synthesis of 11 included studies indicated that high-level RDW was associated with mortality (HR = 1.14, 95% CI 1.09–1.20, Z=5.78, *P*<.001). However, the potential heterogeneity (*I^2 =^* 80%, *P*_heterogeneity_ <.001) was obvious (7), and the meta-analysis lacked the mortality predicting ability, such as the sensitivity and specificity.

In our meta-analysis, the study search was performed through strict screening between Jan. 1, 2000, and May 30, 2022, and all of the studies were published in English. The pooled sensitivity was 0.81 (95% CI 0.73–0.86), and the pooled specificity was 0.65 (95% CI 0.54–0.75), respectively, and had the equivalent predicted value. To evaluate the RDW diagnostic value for predicting mortality in sepsis, SROC and the Fagan test were performed. The SROC curve indicated that the AUC was 0.81 (95% CI 0.77–0.84) with moderate predicted value. Several previous studies indicate the RDW for the prediction of mortality in adult sepsis patients. Wang, A.Y. et al. (48) show that the AUC of RDW in predicting mortality was 0.63 (95% CI 0.52–0.74); however, the included cases were elderly people whose age was more than 65, and the number of included cases was small. Another study (49) showed that RDW was a significant independent prognosis factor of 30-day mortality for sepsis patients, but the AUC of ROC was just 0.66 (0.59–0.73), which was inferior to our meta-analysis. Furthermore, we explored the likelihood ratios and posttest probabilities of RDW for the diagnostic value in predicting mortality, and the result of the Fagan test indicated that, given a pretest probability of 50%, the posttest probability for a positive test result was 70%. Likewise, there was a posttest probability of 23% for a negative test result.

Although this meta-analysis had a useful predicted value of mortality in adult sepsis patients, some limitations were inevitable. First, the research had certain heterogeneity, and the heterogeneity of combined sensitivity and specificity were 96.68 (95% CI 95.95–97.40) and 99.67 (95% CI 99.64–99.71), respectively. We used meta-regression to explore the origin of the heterogeneity, and the results showed that reference description, prospective design, and the blinded interpretation were responsible for the heterogeneity. The main reasons may be as follows (1): Discrepancy references of Sepsis-2 and Sepsis-3 could be responsible for the heterogeneity of reference description, which was consistent with our sensitivity analysis (2). Blindness methods lacked in most studies, and this may be the main reason for heterogeneity of blinded interpretation. (3) For reasons of heterogeneity of prospective design, different research designs from all the included studies (prospective or retrospective design) may be the cause of heterogeneity. Second, the cutoff values of RDW were not consistent, and the cutoff value of RDW in three included studies were not mentioned. This situation may have a negative influence on the diagnostic combined sensitivity and specificity.

In conclusion, RDW is a helpful marker for prediction of mortality in adult sepsis patients. In view of sepsis being an extremely complex syndrome with complex pathophysiological mechanisms, RDW should not be considered as the single definitive test for predicting mortality of sepsis patients, and other useful factors, such as medical history, physical examination, and pathogenic microorganism tests, should be taken into consideration during the clinical process.

## Data availability statement

The datasets presented in this article are not readily available. Because the nature of this research, participants of this study did not agree for their data to be shared publicly, so supporting data is not available. Requests to access the datasets should be directed to crazywu2007@126.com.

## Author contributions

HW had the idea and designed this meta-analysis, and undertook literature search, collection, analysis and writing of this manuscript. BL undertook the data statistical work and the preparation of statistical chart. TJ, JH were responsible for searching relevant literatures. TC and KM had full access to all the data in the study, and took responsibility for the integrity of the data and the accuracy of the data analysis. All authors contributed to the article and approved the submitted version.

## Funding

The funds for research design, data collection and data analysis in this study from the internal medicine research fund of Affiliated Huadu Hospital, Southern Medical University (People’s Hospital of Huadu District). Fund Number: 2020A01. Construction of Major Subject of Affiliated Huadu Hospital, Southern Medical University (YNZDXK202201, 2022-2025).

## Acknowledgments

We acknowledge the support from the Internal Medicine Research Fund of Affiliated Huadu Hospital, Southern Medical University (People’s Hospital of Huadu District).

## Conflict of interest

The authors declare that the research was conducted in the absence of any commercial or financial relationships that could be construed as a potential conflict of interest.

## Publisher’s note

All claims expressed in this article are solely those of the authors and do not necessarily represent those of their affiliated organizations, or those of the publisher, the editors and the reviewers. Any product that may be evaluated in this article, or claim that may be made by its manufacturer, is not guaranteed or endorsed by the publisher.
